# The Middle Miocene Ape *Pierolapithecus catalaunicus* Exhibits Extant Great Ape-Like Morphometric Affinities on Its Patella: Inferences on Knee Function and Evolution

**DOI:** 10.1371/journal.pone.0091944

**Published:** 2014-03-17

**Authors:** Marta Pina, Sergio Almécija, David M. Alba, Matthew C. O'Neill, Salvador Moyà-Solà

**Affiliations:** 1 Institut Català de Paleontologia Miquel Crusafont, Universitat Autònoma de Barcelona, Cerdanyola del Vallès, Barcelona, Spain; 2 Department of Anatomical Sciences, Stony Brook University School of Medicine, Stony Brook, New York, United States of America; 3 NYCEP Morphometrics Group; 4 Dipartimento di Scienze della Terra, Università degli Studi di Torino, Torino, Italy; 5 ICREA at Institut Català de Paleontologia Miquel Crusafont and Unitat d'Antropologia Biològica (Dept. BABVE), Universitat Autònoma de Barcelona, Cerdanyola del Vallès, Barcelona, Spain; University of Florence, Italy

## Abstract

The mosaic nature of the Miocene ape postcranium hinders the reconstruction of the positional behavior and locomotion of these taxa based on isolated elements only. The fossil great ape *Pierolapithecus catalaunicus* (IPS 21350 skeleton; 11.9 Ma) exhibits a relatively wide and shallow thorax with moderate hand length and phalangeal curvature, dorsally-oriented metacarpophalangeal joints, and loss of ulnocarpal articulation. This evidence reveals enhanced orthograde postures without modern ape-like below-branch suspensory adaptations. Therefore, it has been proposed that natural selection enhanced vertical climbing (and not suspension per se) in *Pierolapithecus catalaunicus*. Although limb long bones are not available for this species, its patella (IPS 21350.37) can potentially provide insights into its knee function and thus on the complexity of its total morphological pattern. Here we provide a detailed description and morphometric analyses of IPS 21350.37, which are based on four external dimensions intended to capture the overall patellar shape. Our results reveal that the patella of *Pierolapithecus* is similar to that of extant great apes: proximodistally short, mediolaterally broad and anteroposteriorly thin. Previous biomechanical studies of the anthropoid knee based on the same measurements proposed that the modern great ape patella reflects a mobile knee joint while the long, narrow and thick patella of platyrrhine and especially cercopithecoid monkeys would increase the quadriceps moment arm in knee extension during walking, galloping, climbing and leaping. The patella of *Pierolapithecus* differs not only from that of monkeys and hylobatids, but also from that of basal hominoids (e.g., *Proconsul* and *Nacholapithecus*), which display slightly thinner patellae than extant great apes (the previously-inferred plesiomorphic hominoid condition). If patellar shape in *Pierolapithecus* is related to modern great ape-like knee function, our results suggest that increased knee mobility might have originally evolved in relation to enhanced climbing capabilities in great apes (such as specialized vertical climbing).

## Introduction

The partial hominoid skeleton IPS 21350 from the locality of Barranc de Can Vila 1 [1]–[8], situated in the local stratigraphic series of Abocador de Can Mata (ACM/BCV1; els Hostalets de Pierola, Vallès-Penedès Basin, NE Iberian Peninsula), constitutes the holotype (and so far only known individual) of *Pierolapithecus catalaunicus*. With an estimated age of 11.9 Ma (late Aragonian, Middle Miocene) [9], [10], *Pierolapithecus* is the oldest undisputed extinct member of the great-ape-and-human clade—i.e., the Hominidae [1], [2], [6], [7], [11].

IPS 21350 comprises more than 80 bones or bone fragments, including the splanchnocranium, key regions of the wrist and ankle complexes, a clavicle, vertebrae and ribs, as well as fragmentary remains of the pelvis and an almost complete patella [1]. The preserved anatomy provides strong evidence of advanced orthograde postures as compared to previous apes [1], [5], [8], although the fragmentary pelvic remains indicate only slight differences from *Proconsul* [8], stressing the mosaic nature of the postcranial skeleton evolution in Miocene apes [12]. At the same time, hand length proportions and phalangeal anatomy indicate that modern ape-like below-branch suspensory adaptations are lacking. In particular, the hand displays only a moderate length and phalangeal curvature ([2], [4], [13] but see [14], [15] for a different interpretation), the metacarpophalangeal joints are dorsally oriented, and the pollical distal phalanx is long and wide at the base relative to the distal phalanges of the lateral rays [4], [16]. These features indicate that *Pierolapithecus*—as in other Miocene apes—relied significantly on above-branch palmigrady with a thumb-assisted grasping during arboreal locomotion [12], [17]. Moreover, and as in extant great apes, the triquetrum was distally situated on the wrist, showing a crevice for attachment of a meniscus instead of an articular facet for the ulnar styloid process [1]. The combination of an orthograde body plan and the loss of ulnocarpal articulation (i.e., enhancing ulnar deviation of the hand) with no specific below-branch adaptations suggests that vertical climbing—and not suspension per se*—*might have been the primary target of natural selection in *Pierolapithecus*, since it is the only other common behavior to the hominoid crown group [1], [2]. The mosaic nature of the Miocene ape postcranial skeleton should prevent straightforward locomotor reconstructions based solely on isolated anatomical parts in these fossil forms. Instead, different anatomical regions should be considered together (when possible) to more accurately reconstruct their locomotor adaptations. However, although hind limb long bones of *Pierolapithecus* are not preserved (other than shaft fragments), the morphology of its preserved patella (IPS 21350.37) can potentially provide hints of its knee function, as previous studies have shown for other Miocene taxa [18]. Here we provide a detailed description of the patella from the holotype of *Pierolapithecus* (IPS 21350) as well as an exhaustive morphometric analysis with selected extant anthropoids and available fossil hominoids. Therefore, the aim of this study is to shed light on the patellar morphology and inferred knee function of *Pierolapithecus catalaunicus*.

## Materials and Methods

The studied specimen (IPS 21350.37) is housed at the Institut Català de Paleontologia Miquel Crusafont (Sabadell, Spain). To compare this specimen with the patellae of other (extant and extinct) anthropoids, four variables were measured following Ward et al. [18]: total proximodistal height of the patella (PD); proximodistal height of the articular surface (PDAS); anteroposterior thickness (AP); and mediolateral breadth (ML). These variables are intended to capture the overall proportions of the patella while being biomechanically meaningful. Measurements were taken using a digital caliper to the nearest 0.1 mm. The individual values for *Pierolapithecus* were compared with the sample of extant anthropoids used by Ward et al. (their Tables 1 and 2) [18], as well as selected fossil hominoid specimens, for which measurements were taken from the literature [18]–[20]. In all cases, only adult specimens for whom all measurements were available were included in the analyses. The fossil hominoid sample included: KPS PT3 and KPS PT4 (*Proconsul heseloni*) [18]; KNM-RU 17382 (*Proconsul nyanzae*) [18]; KNM-BG 15535 (*Nacholapithecus kerioi*, referred to *Kenyapithecus* in [18]); BAC 122 (*Oreopithecus bambolii*, measured by S.A. from a cast: PD  = 22.2 mm, PDAS  = 19.9 mm, AP  = 8.9 mm, ML  = 23.0 mm); and KNM-MB 24738 (*Equatorius africanus*) [19].

**Table 1:** Ordinary least squares (OLS) and phylogenetic generalized least-squares (PGLS) allometric regressions for mediolateral breadth of the patella (ML) relative to body mass (BM) and patellar size (GM).

OLS	Intercept	s.e.	Slope	s.e.	95% CI	F	p-value	Adj R
ML vs BM	1.864	0.064	0.360	0.024	0.305–0.414	229.875	<0.001	0.962
ML vs GM	−0.478	0.222	1.243	0.085	1.047–1.439	214.469	<0.001	0.960

PGLS	Intercept	s.e.	Slope	s.e.	95% CI	t -value	p-value	Adj R	λ	DF
ML vs BM	1.815	0.067	0.376	0.025	0.318–0.433	15.037	<0.001	0.960	0.000	8
ML vs GM	−0.348	0.192	1.190	0.075	1.020–1.360	15.930	<0.001	0.970	1.000	8

Regressions were derived in the extant non-human anthropoids sample (8 species: 5 monkeys, 3 great apes) using female individuals data set (sex-pooled humans were not included in the analyses; see text for further explanation).

Abbreviations: ML, mediolateral breadth of the patella; BM, body mass; GM, geometic mean based on the four lengths measured on the patella; s.e., standard deviation; CI, confidence interval; Adj, adjusted; DF, degrees of freedom.

a p<0.05, based on *t*-statistic for the coefficient.

**Table 2:** Results of the Between Groups Principal Components Analysis (BgPCA) based on patellar measurements.

	BgPC1	BgPC2
**% variance**	61.371	30.285
**Variable loadings^a^**		
PD	−0.18	**0.61**
PDAS	0.34	0.38
AP	**−0.73**	−0.41
ML	**0.57**	**−0.57**

Abbreviations: bgPC, between-group principal component; PD, total proximodistal height of the patella; PDAS, proximodistal height of the articular surface; AP, anteroposterior thickness; ML, mediolateral breadth. ^a^ Each original variable was size-adjusted by the geometric mean (GM) of the four variables and log-transformed (using natural logarithms) prior incorporation into the analysis. The variables with absolute loadings of 0.5 or more are marked in bold. Only the two first bgPC axes provided meaningful discrimination and are therefore shown.

For shape comparisons, linear dimension were divided by overall patellar size, which was approximated by the geometric mean (GM) of the four original lengths. Scaling the patellar linear dimensions by the GM gives individual dimensionless Mosimann shape ratios that are independent of the remaining sample (unlike residuals derived from regressions) [21], [22]. Comparisons of patellar size (GM) and shape (Mosimann variables) were depicted by means of boxplots. Further, major patterns of patellar shape variation between extant anthropoids and fossil hominoids were summarized by means of a principal components analysis (PCA) performed on the covariance matrix of the taxa means. Individual PC scores were computed and plotted a posteriori in order to show variation within extant anthropoids. The method, known as between-group PCA (bgPCA), is extensively described elsewhere [23]. Shape variables were log-transformed (using natural logarithms) before being introduced into the analysis. Statistical differences between the bgPC scores obtained (bgPC1 and bgPC2 in our case) from our extant sample of primates were inspected by means of analyses of their variance (ANOVA), as well as multivariate analyses of variance (MANOVA; to inspect both principal axes together), and their associated Bonferroni post hoc multiple comparisons. All shape analyses were performed with the statistical packages *SPSS* v 15 and *PAST* v 2.15.

Patellar mediolateral breadth (ML) has been found to scale with body mass (BM) in non-human hominoids [24]. We inspected the scaling of ML against BM and GM in our sample of non-human anthropoid primates by means of phylogenetic generalized least-squares (PGLS) regressions of the log-transformed, sex-specific means. The regression coefficients and the error term are all computed by means of maximum likelihood [25], with phylogenetic signal [26], [27] incorporated into the error term. The degree of phylogenetic signal is given by λ, which varies between values of 0 (no signal) and 1 (strong signal) [26], [27]. All PGLS regressions results are based on female species means; the male results were similar and therefore are not shown. PGLS regression statistics were calculated using the ‘base’ and ‘caper’ libraries of R (v 2.9; R Development Core Team, 2008). The consensus topology and branch lengths for the extant primate sample were taken from the 10 k Trees website (v3) [28].

## Results

### Description and Measurements

**Figure 1:**
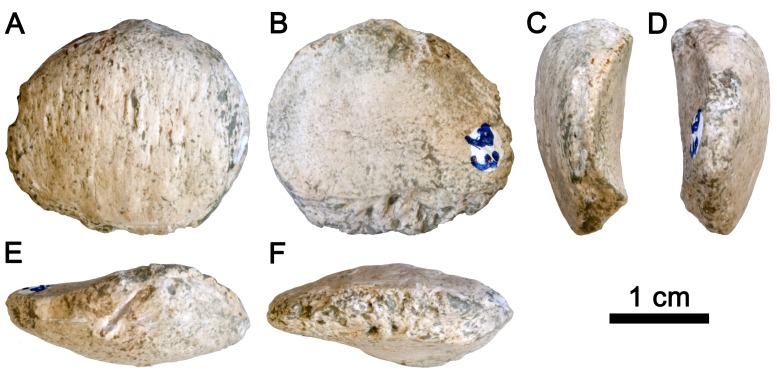
Patella of *Pierolapithecus catalaunicus*. IPS 21350.37 is shown in anterior (A), posterior (B), lateral (C), medial (D), proximal (E) and distal (F) views.

The left patella IPS 21350.37 (Fig. 1) is well preserved, except for very minor damage on its proximal (caused during excavation; Fig. 1B,E) and medial (Fig. 1B,D) portions, as well as some superficial abrasion on the distal end. However, this very minor abrasion did not preclude taking complete measurements of the relevant dimensions. IPS 21350.37 extends more mediolaterally (ML  = 24.9 mm) than proximodistally (PD  = 21.9 mm), and exhibits moderate anteroposterior thickness (AP  = 9.7 mm) that slightly wedges distally. The anterior side displays a rough surface on the proximal half for the insertion of the muscles vastus lateralis, medialis, intermedius and rectus femoris (i.e., quadriceps muscle group) and their associated tendons. The posterior side is almost completely covered by the articular surface for the femoral patellar groove. The lateral portion of the articular surface is larger than the medial one, and the contour of the latter is slightly damaged. The proximodistal height of the articular surface can be reliably measured (PDAS  = 17.1 mm). Running through the distal edge, a rough area for the attachment of the patellar ligament is evident and courses medially. This attachment is slightly abraded (Fig. 1F).

### Mosimann Shape Variables

**Figure 2:**
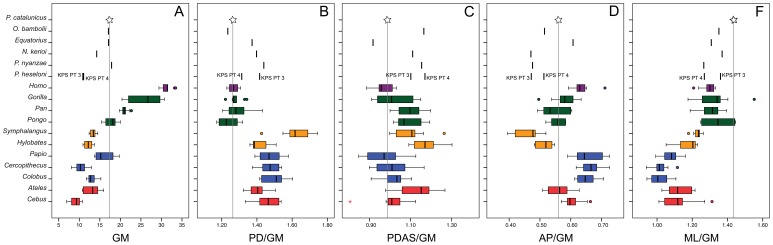
Boxplots representing patellar size (GM) and Mosimann shape variables. A, patellar size (GM); B–F, shape variables standardized by GM based on the four original variables. Vertical lines represent the median, boxes the interquartile range (between 25th and the 75th percentiles), whiskers the extreme values, circles the outliers and asterisks the extreme outliers. Colors indicate the major taxonomic groups: blue, cercopithecoids; red, platyrrhines; orange, hylobatids; green, great apes; purple, humans. *Pierolapithecus* is highlighted by a star and the grey vertical line in every boxplot is meant to facilitate the visual comparison with the remaining taxa.

**Figure 3:**
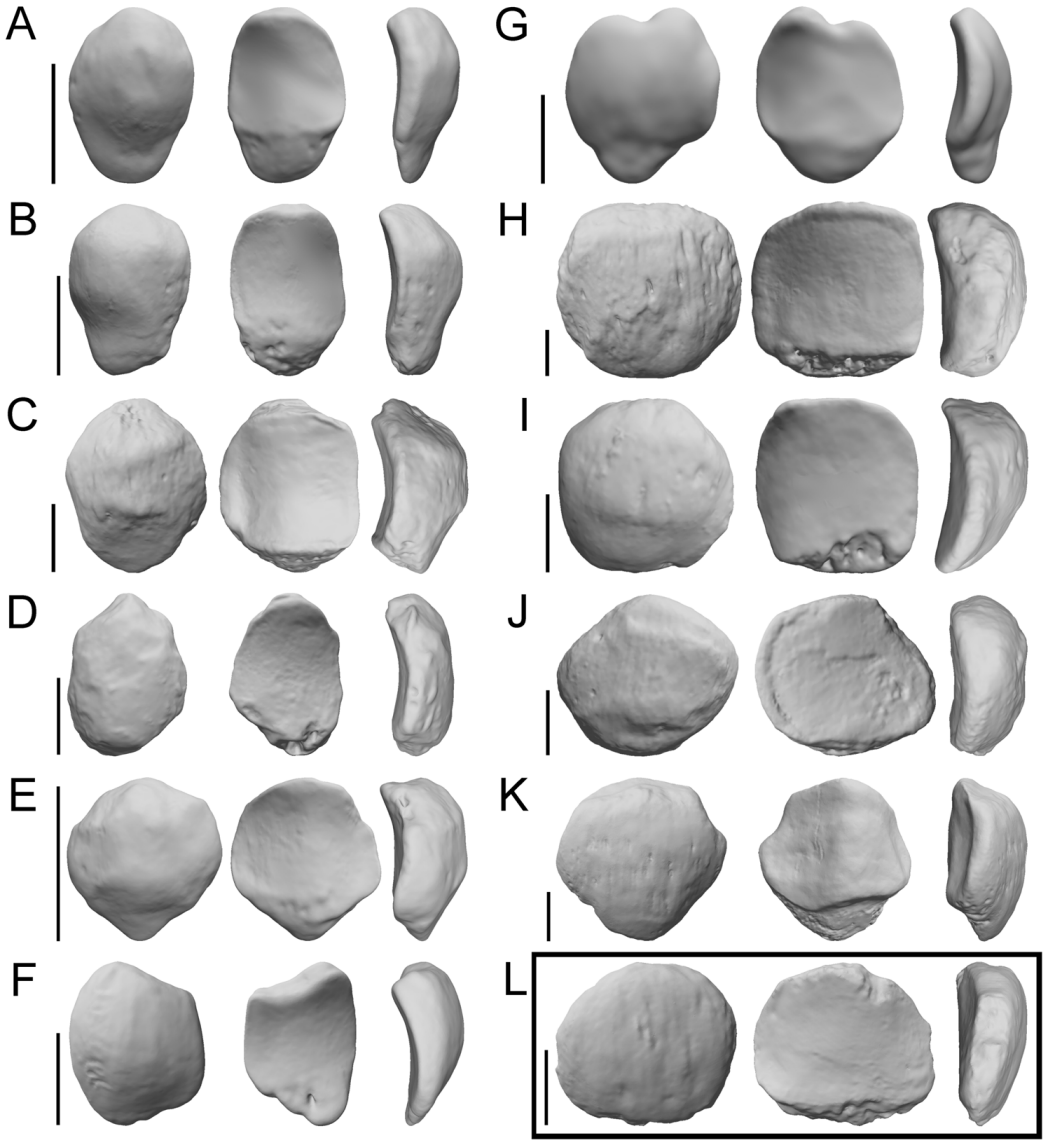
Patellar 3D virtual models of selected extant anthropoid genera and *Pierolapithecus*. Anterior (left), posterior (middle), and side (right) views of: A, *Cercopithecus* (right, reversed); B, *Colobus* (right, reversed); C, *Papio* (right, reversed); D, *Ateles* (right, reversed); E, *Cebus* (left); F, *Hylobates* (left); G, *Symphalangus* (right, reversed); H, *Gorilla* (left); I, *Pan* (right, reversed); J, *Pongo* (right, reversed); K, *Homo* (left); and L, *Pierolapithecus* (IPS 21350.37, left). Patellae were scanned by a superficial laser scan (provided by P. Ibáñez). Then 3D models were created and scaled to the same proximodistal size for a better visualization. Scale bars equal 1 cm.

The range of variation of the patellar size (GM), as well as the Mosimann shape variables for the different extant genera and fossil individuals, are depicted in Figure 2 by means of boxplots (see Fig. 3 for patellar morphological comparisons). Regarding the overall patellar size (GM), African apes and, especially, humans have the largest patellae (Fig. 2A). Apart from *P. heseloni* and *Nacholapithecus*, which are similar to hylobatids and monkeys (platyrrhines and cercopithecoids), the rest of Miocene apes, including *Pierolapithecus*, have patellae of intermediate size between the monkey-hylobatid group (except *Papio*) and African ape-human group, overlapping with the ranges of orangutans and baboons.

Monkeys and hylobatids exhibit proximodistally longer patellae than extant great apes and humans (Fig. 2B). That of *Symphalangus* is exceptionally large, its lower non-interquartile range overlapping only with the upper range of cercopithecoid monkeys, but not with that of *Hylobates*. As for extant great apes, only the uppermost range of *Pan* overlaps with that of monkeys and *Hylobates*. *Pierolapithecus* (similarly as *Oreopithecus*) falls within the interquartil range of all great apes and humans, while the rest of Miocene apes exhibit slightly proximodistally longer patellae, falling in an intermediate position between monkeys-*Hylobates* and great apes.

For PDAS, differences between genera are less clear (Fig. 2C). Although most ranges overlap, humans, cercopithecoids and *Cebus* show proximodistally shorter articular surfaces than *Pan, Pongo*, hylobatids and *Ateles*. Gorillas display a wide range, overlapping with the interquartile ranges of the remaining great apes and all monkeys. *Hylobates* shows the highest values of PDAS, closely followed by *Ateles*. *Pierolapithecus* overlaps with humans, gorillas and monkeys (although only slightly with the lowermost range of *Ateles*). *Oreopithecus* shows one of the lowest values for this ratio, conversely to the rest of Miocene apes, whose ratios overlap with those of *Hylobates*, *Ateles* and the uppermost part of the interquartile range of great apes and *Symphalangus*.

In contrast, marked differences are observed concerning anteroposterior thickness (Fig. 2D). Hylobatids display the thinnest patellae, whereas cercopithecoids and humans show the opposite condition. Platyrrhines and great apes display a more intermediate position, as *Pierolapithecus*. *Oreopithecus*, *Nacholapithecus* and *Proconsul* show slightly thinner patellae than great apes, overlapping with the lowest range of *Pan*. *Equatorius* is more similar to humans and cercopithecoids, although it also falls in the range of gorillas. Finally, cercopithecoids display the narrowest patellae (Fig. 2F), followed by platyrrhines, hylobatids, and great apes and humans. Miocene apes overlap with the ranges of the last two groups, showing one specimen of *P. heseloni* (KPS PT 4) and *P. nyanzae* the lowest values of ML among fossils. *Equatorius*, *Oreopithecus*, the other individual of *P. heseloni* (KPS PT 3) and *Nacholapithecus* show intermediate values for fossils, being *Pierolapithecus* the specimen with the broadest mediolateral length of the patella.

### Size Scaling of Patellar Mediolateral Breadth

**Figure 4:**
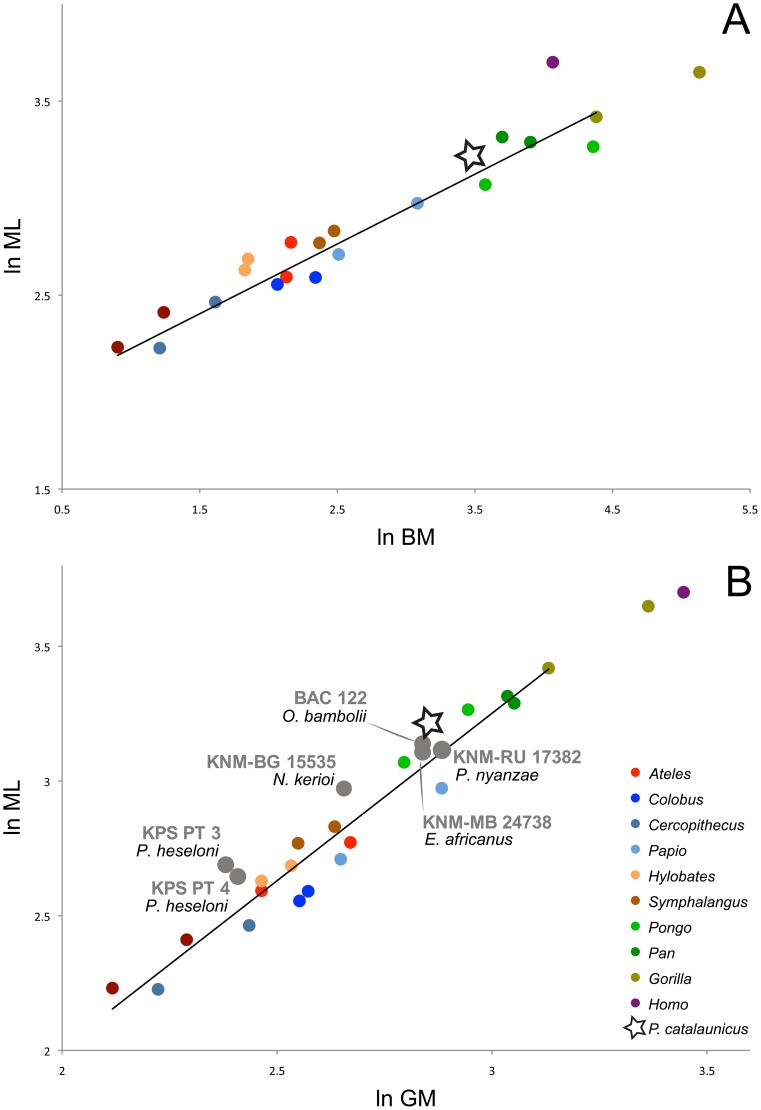
Allometric bivariate plots. A, mediolateral breadth (ML) vs. body mass (BM); B, ML vs. patellar size (GM). The OLS and PGLS allometric regression equations are reported in Table 1; black line denotes female means of non-humans primates OLS regression (see text for further explanation). Because of the isometric relationship between ML and BM, the former can be used as a surrogate of BM (see text).

Regression results are given in Figure 4 and Table 1. For both the ML vs. BM and ML vs. GM, the results are near expectations based on isometric dimensional scaling. Mediolateral patellar breadth exhibits a strong correlation with BM, and scales with a slope of 0.376±0.025. Because λ = 0.000, the 95% confidence intervals (CI) was calculated using a t distribution for small samples (DF  = 8, t = 2.306, α = 0.05), yielding a slope CI of 0.318–0.433, which overlaps the isometric expectation of 0.333. ML also exhibits a strong correlation with GM, and scales with a slope of 1.190. The λ = 1.000 complicates use of standard statistical tables in this instance. However, it is likely that this scaling pattern shows a significantly positive allometry by a small margin (est. 95% CI 1.020–1.360), based on an isometric expectation of 1.000. Therefore, the above-explained differences between hominids and the hylobatid-monkey group in the Mosimann ratio ML/GM (Fig. 2F) may be due to scaling effects.

### Between-Group Principal Components Analysis

**Figure 5:**
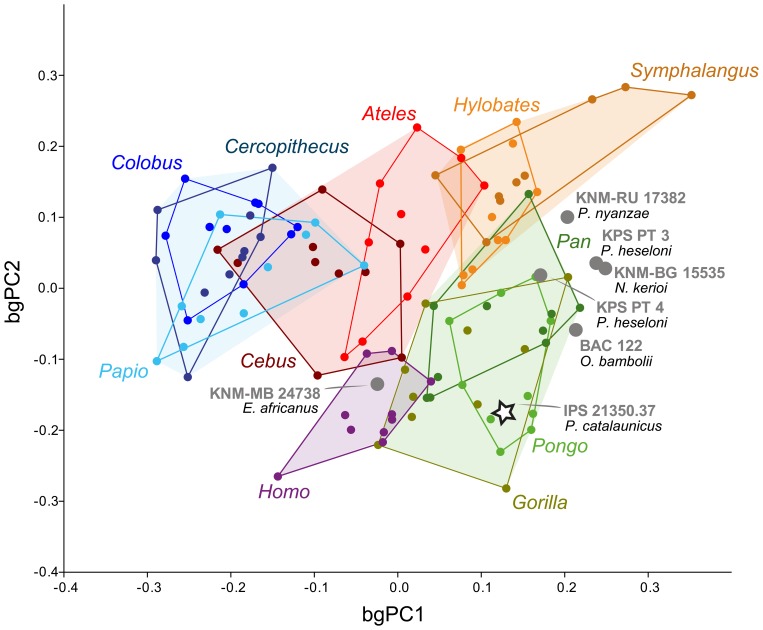
Between Groups Principal Components Analysis (BgPCA) performed on extant taxa and individual fossil patellae. The first two axes explain up to 91.6% of the total variance (BgPC1, 61.4%; BgPC2, 30.3%). Major taxonomic groups are indicated by colors as follows: blue, cercopithecoids; red, platyrrhines; orange, hylobatids; green, great apes; purple, humans. *Pierolapithecus* is highlighted by a star. See Material and methods and Table 1 for more details.

Most of the patellar shape variation (91.6%) among extant and fossil taxa is explained by the two first between-group principal components (bgPCs; Fig. 5; Table 2). bgPC1 (61.4% of variance) is highly correlated with positive values of mediolateral patellar breadth (ML) and especially negative values of anteroposterior patellar thickness (AP). This axis completely separates apes from cercopithecoids. However, platyrrhines and humans overlap on this axis and occupy an intermediate position between cercopithecoids and apes (overlapping with both). Differences in bgPC1 scores between taxa are statistically significant (F = 50.378, p<0.001; see Table S1 for specific differences). These results highlight the fact that monkeys and, especially, cercopithecoids have anteroposteriorly thicker and mediolaterally narrower patellae than extant great apes (see also Fig. 3). *Symphalangus* exhibits the extreme condition for hominoids, being statistically different from the remaining taxa except for *Hylobates* (p = 1.000; Table S1). Conversely, modern humans, although in the range of platyrrhines, show significant differences with all cercopithecoids and extant ape genera (p<0.05; Table 2). bgPC2 (30.3% of variance) is highly correlated with positive values of proximodistal patellar length (PD) and negative values of mediolateral breadth (ML). bgPC scores for this axis also show statistical differences among genera (F = 14.882, p<0.001). Cercopithecoids, platyrrhines and hylobatids display overall significant differences from extant great apes and humans (p<0.05; Table S1). Thus, although there is overlap in the bgPC2 ranges of all great apes with those of hylobatids and monkeys, the two latter groups show relatively longer and narrower patellae than great apes and humans (see also Fig. 3). Again, *Symphalangus* shows the extreme positive values along bgPC2, by having the highest relative patellar proximodistal length and lowest anteroposterior thickness. The MANOVA results reveal that, when the two first bgPC axes are considered together, differences are also statistically significant. All cercopithecoid taxa are statistically different from the ape taxa, and *Symphalangus* shows differences with the remaining primate genera to the exception of *Hylobates* (p<0.001). Modern humans display differences with apes and cercopithecoids (p<0.05), but not with platyrrhines (p = 1.000; Table 2). Thus, to some degree, patellar shape differences (as identified by our bgPCA) relate to phylogeny. Great apes are more similar among them than to hylobatids, cercopithecoid taxa are more similar to each other than to great apes, and this is also the case of platyrrhine taxa. However, concerning bgPC1 (the axis that explains the highest amount of variance), cercopithecoids are more distinct from hominoids than are platyrrhines (intermediate between both).

Most fossil apes (the two species of *Proconsul*, *Nacholapithecus* and *Oreopithecus*) fall close in the bgPC1-bgPC2 morphospace, highly overlapping with *Symphalangus* and great apes (mainly the specimens KPS PT 4, BAC 122 and KNM-RU 17382) for bgPC1. These fossil apes occupy a central position along bgPC2, overlapping with extant apes and monkeys. BAC 122 (*Oreopithecus*) shows the lowest values among the above-mentioned Miocene apes, and KNM-RU 17382 (*P. nyanzae*) the highest. Overall, the patella of these Miocene apes is relatively thin anteroposteriorly and wide mediolaterally, in the uppermost range or just above the extant great ape range (bgPC1), and in the upper range of great apes for bgPC2 (by discounting one *Pan* outlier), but fully within the monkey range for the latter axis. Two fossil ape patellae depart from the others: KNM-MB 24738 (*Equatorius*) and IPS 21350.37 (*Pierolapithecus*). They show both lower bgPC1 (especially *Equatorius*) and bgPC2 values than the remaining Miocene apes. When both bgPC axes are inspected together, to the exception of KPS PT4 (*P. heseloni*, which overlaps with *Pan* and is also close to *Pongo*), the other *Proconsul, Nacholapithecus* and *Oreopithecus* specimens fall in a unique region of the morphospace. *Equatorius* shows its closest affinities with modern humans, and *Pierolapithecus* overlaps with *Pongo* and *Gorilla*.

## Discussion

### Patellar Shape and Function in Extant Anthropoids

Differences in patellar morphology between monkeys and hominoids (especially great apes) have been previously noted on the basis of the external dimensions used herein (PD, PDAS, AP, and ML): monkeys exhibit proximodistally taller, anteroposteriorly thicker and mediolaterally narrower patellae than great apes (Figs. 3 and 5) [18], [20], [29]. These external dimensions have been used to make functional inferences for Miocene apes [18]. In particular, Ward and colleagues [18] concluded that differences in external proportions of the patella between monkeys and apes indicate biomechanical differences in their knee function, related to bone stresses. However, it should be noted that only few mechanical models of the non-human primate knee joint have considered the coronal plane [30], [31], [32], and this is not the case of the above-mentioned study on Miocene apes. Taking that into account, the biomechanical notes that follow are only meant to discuss patellar shape differences between monkeys and apes in the light of available mechanicals models of the knee—restricted to the sagittal plane—that have been previously used to infer hind limb function in Miocene apes.

Our results agree with a previous study [24] according to which, in non-human hominoids, the mediolateral breadth of the patella scales with geometric isometry to body mass (BM), and further indicate that this assertion holds not only for apes, but for monkeys as well. Humans, in contrast, are clear outliers in this regression, due to their bipedal locomotor behavior (Fig. 4A). Since no significant grade shifts between monkeys and apes (only hylobatids are slightly upshifted) have been found [18], [24] (see also Fig. 4A), it has been hypothesized that mediolateral patellar breadth is relatively unaffected by the type of locomotion [18], further providing a good surrogate of BM irrespective of phylogenetic constraints.

However, PD and AP seem to display a strong functional signal [18], [29]. Our results, in agreement with previous work [18], show that anteroposterior thickness of the patella is relatively higher in cercopithecoids than in platyrrhines and apes, respectively (Fig. 2D); whereas PD is higher in monkeys and hylobatids (displaying *Symphalangus* the proximodistally highest patella) than in great apes (Fig. 2B). This latter fact might be related to the presence of a large non-articular surface, the apex, in the patellae of monkeys and hylobatids (Fig. 3). Therefore, PD and AP mainly differentiate monkeys and great apes. Both parameters have been previously associated with the increase of the moment arm of the quadriceps tendon-ligamentum patellae about the knee joint [18], [33]. In the case of AP, a thicker patella mainly separates the ligamentum patellae from the center of rotation of the knee in the sagittal plane, changing the angle of action of the quadriceps muscle mainly during flexed knee positions as well as increasing the moment arm of the muscle. Regarding PD, the greater length of the patella (including the apex) increases the lever arm of the quadriceps muscle from a flexed posture of the knee, thus enhancing the torque or rotational force of the joint [18], [34]. Therefore, the higher moment arms generated by a large proximodistal and thick anteroposterior patellae about the knee joint probably favor the forceful extension of that joint from fully-flexed positions [18], [33], [35], [36]. Although not mentioned in previous studies, a higher moment arm also implies a lower angular velocity [37], hindering a quick extension of the knee mainly during leaping. In this regard, further work is needed to solve this dichotomy and better understand the biomechanics of the primate knee and its relationship with patellar morphology. Thus, when AP and PD are assessed within a positional context, it can be observed that primates which rely on leaping and galloping (with predominant excursions of the joint from a full-flexed knee to extended positions) display higher values of these two parameters (Figs. 2 and 5) [18], [29]. The proximodistally short and anteroposteriorly thin patellae of great apes have been associated with a more versatile knee, with a wider range of positions and no habitual full flexion of the knee [18], [29]. The locomotor repertoire of these taxa (probably related to their large body mass) does not include frequent leaping or galloping. Instead they practice more frequently orthograde behaviors, such as vertical climbing, below-branch suspension, clambering and bridging (e.g., [18], [38], [39]). Since great apes show fully-flexed knee positions in a notably lower frequency than monkeys (only orangutans clearly full-extend the knee during arboreal bipedalism) [18], [39]–[42], so that their shorter anteroposteriorly and proximodistally patellae might reflect these different biomechanical demands relative to non-hominid anthropoids (i.e., lower moment arms in the knee).

Furthermore, African apes and orangutans differ in type of locomotion and frequency of arboreal behaviors [42]–[45]. The former are characterized by the practice of knuckle-walking, which implies an assemblage of specific adaptations [38], [46], [47]. In contrast, orangutans are more arboreal, and mostly rely on below-branch suspension and clambering for traveling horizontally [40], [41], [48], [49]. Apart from some degree of suspension, vertical climbing (upright trunk progression on arboreal supports employing hind limb propulsion and hands and feet grasping) seems to be the common locomotor behavior among all extant apes [40], [50]. Hylobatids, and especially *Symphalangus* (which employ vertical climbing even more often than great apes [50]), employ less abducted hind limb positions than the latter during vertical climbing [41], [50]. It is noteworthy that African apes and orangutans practice vertical climbing in different frequencies, and that there are also some differences in the hind limb use, since in orangutans the knee is less flexed and more extended, and the hip is more flexed and abducted, than in African apes [40], [41], [48]. Likewise, orangutans possess a larger mass of knee flexor muscles relative to the extensors, thus favoring the rotation and flexion of the knee as well as a wider variety of postures at this joint [49]. However, these differences are not reflected in the overall proportions of the patella as captured by our analyses (Figs. 2 and 5). Nonetheless, African apes display a trapezoidal patellar surface in the distal epiphysis of the femur (Fig. 3), which might reflect a decreased mobility of the knee joint compared to orangutans [20]. Thus, although further studies are needed in this regard, the African ape configuration might be slightly derived among extant great apes, being potentially related to an increase in knee stability during knuckle-walking. In fact, orangutans show a greater capability of knee rotation, as well as a higher range of motion of their joints, when compared to African apes [40], [49].

### Inferences on Knee Function and the Evolution of *Pierolapithecus* and Other Miocene Apes

In the above-mentioned regards, the patella of *Pierolapithecus* is essentially similar to that of great apes (and especially orangutans and gorillas; Figs. 2, 3 and 5). The comparable patellar morphology of *Pierolapithecus* and great apes suggests a similar biomechanical loading regime (and associated joint positions), with no habitual and stereotyped flexion-extension of the knee joint. This positional hypothesis is compatible with the orthograde body plan inferred for *Pierolapithecus* on the basis of its thorax morphology [1]. In this taxon, the lack of extant ape-like specific adaptations to below-branch suspensory behaviors (e.g., moderate hand length and phalangeal curvature), combined with its orthograde body plan and loss of ulnocarpal contact, led previous authors to suggest that enhanced vertical climbing capabilities (compared to previous apes) was the main target of natural selection [1], [2], [4], [13]. Previous inferences of above-branch palmigrady for *Pierolapithecus*, based on hand morphology (e.g., dorsally oriented metacarpo-phalangeal joints) [1], [4], [13], are a priori less consistent not only with orthogrady, but also with the great ape-like patellar morphology observed for this taxon in our analyses. However, the above-branch quadrupedalism displayed by *Pierolapithecus* probably has no modern analog, due to its tailless condition and powerful-grasping (thumb-assisted) capabilities inferred for this and other Miocene apes [1], [4], [13], [16], [51]. *Pierolapithecus* also exhibits a pelvic morphology similar to *Proconsul*, but with a slightly more marked lateral flaring of the ilia [8]. Unfortunately no femoral remains are available for this taxon, although those available for other Miocene hominoids have been found to share a similar proximal shape to each other [52]. This fact may suggest similar and unique hip biomechanics for most Miocene apes, which would display (like in *Proconsul*) a mosaic postcranial morphology, combining in the case of *Pierolapithecus* an orthograde body plan with above-branch palmigrady, great ape-like knee function and hip joint with increased ape-like mobility (e.g., [4], [8], [12], [53]).

In evolutionary terms, our results suggest that cercopithecoids might display, concerning the anteroposterior dimension, the most derived patella among anthropoids (Figs. 2 and 5). However, great apes show somewhat anteroposteriorly thinner patellae than monkeys, although thicker than those of the fossil taxa (Fig. 5; Table 2). Ward et al. [18] proposed that the patellar morphology of stem hominoids such as *Proconsul* and *Nacholapithecus* would be representative of the primitive hominoid condition—i.e., proximodistally higher, anteroposteriorly thinner and mediolaterally narrower patellae compared with those of extant great apes. Therefore, the quadriceps muscle mechanical advantage may have increased in the course of hominoid evolution, but never attaining the extreme values of cercopithecoids. This fact might be related to the more varied locomotor repertoire of great apes than that of monkeys, being *Pierolapithecus* similar to the former group in this regard. The external morphology of the patella of *Equatorius*, in turn, is closer to that of African apes, and even to that of modern humans (Figs. 2, 3 and 5). This fact might be explained by the pronograde, semi terrestrial behaviors inferred for this taxon [54]–[56]. This type of locomotion, to a large extent, would be similar in functional requirements to the locomotor repertoire of chimpanzees and bonobos, which rely on arboreal behaviors more frequently than gorillas. This positional behavior requires a highly versatile knee joint for combining orthograde locomotor behaviors (such as vertical climbing and suspension) with quadrupedalism in both arboreal and terrestrial substrates. In addition to the specimen KPS PT 4 (*P. heseloni*), the patellae of *Pierolapithecus* and *Oreopithecus*—the only widely accepted orthograde taxa among the analyzed fossil apes [1], [5], [57], [58]—are those that most closely resemble great-ape patellae (Figs. 2, 3 and 5), probably exhibiting a versatile knee joint.

Given that the evolution of the locomotor apparatus in apes during the Miocene apparently proceeded in a mosaic fashion (e.g., [6], [12], [13], [58]), and the current decimated diversity of extant hominoids, there should not be surprising that there are no extant locomotor analogs for these extinct taxa [1], [6], [8], [58]. The above-branch quadrupedal component and the lack of specific below-branch suspensory adaptations inferred for *Pierolapithecus* suggest that its great ape-like patellar morphology might be simply attributable to the higher range of knee motion required by orthograde vertical climbing, which would have been probably most similar to that performed by extant great apes (with extended hip joints and flexed knees, and more abducted hind limb positions than in lesser apes) [1], [4], [5], [13], [40], [41]. All extant hominoids share a similar orthograde body plan, suitable for both vertical climbing and below-branch suspensory behaviors (and bipedalism in hominins) [40], [41], [48]. However, the evidence provided by *Pierolapithecus* [1], [2], [4], [8], [13] suggests that the acquisition of suspensory adaptations might have been decoupled from that of vertical climbing (contra [11], [14], [15])—with clear suspensory adaptations not being displayed until the late Miocene by *Hispanopithecus*/*Rudapithecus* (see discussion in [59], but also [13]–[15], [17], [52], [60]–[62]). Concerning the latter taxa, the below-branch suspensory adaptations observed on their femora [52], [60], [61], [63], [64] and other postcranial remains [13]–[15], [17], [60]–[62] lead us to predict, based on our analyses above, that the patella of *Hispanopithecus* (if ever found) would probably resemble those of modern great apes, like in *Pierolapithecus* and *Oreopithecus*.

## Conclusions

Morphometric analyses of the patella of *Pierolapithecus catalaunicus* reveal its closest shape affinities with those of extant great apes. The great ape-like—i.e., proximodistally short, anteroposteriorly thin and mediolaterally broad—patellar morphology displayed by *Pierolapithecus* is functionally related (on the basis of available knee mechanical models) to a wider range of movements at the knee joint [18], [29], thereby suggesting that this fossil great ape probably exhibited a more versatile knee joint than the more stereotyped configuration (full flexion-extension) characteristic of monkeys (especially cercopithecoids). In contrast, the patellae of stem hominoids (such as *Proconsul* or *Nacholapithecus*) differ by being anteroposteriorly more compressed than that of *Pierolapithecus* (a morphology that has been inferred to be the plesiomorphic condition for hominoids). In turn, hylobatids display a patellar morphology intermediate between those of monkeys and great apes (an even autapomorphic to some degree). We hypothesize that, taking into account the lack of specific suspensory adaptations in the orthograde *Pierolapithecus* (which still heavily relied on above-branch quadrupedalism), its great ape-like patellar morphology probably evolved originally in conjunction with orthograde behaviors (other than suspension) requiring increased versatile movements of the knee joint, such as specialized vertical climbing.

## Supporting Information

Table S1 **Significance for the ANOVA and MANOVA multiple post hoc comparisons (Bonferroni method) for scores of bgPC 1 and 2 according to extant primates genera.**
(PDF)
