# Supplementary material for: The Middle Miocene Ape Pierolapithecus catalaunicus Exhibits Extant Great Ape-Like Morphometric Affinities on Its Patella: Inferences on Knee Function and Evolution
Source: PLoS One. 2014 Mar 17;9(3):e91944. doi: 10.1371/journal.pone.0091944 (PMC3956854; doi:10.1371/journal.pone.0091944)
Supplement: Table S1 — Significance for the ANOVA and MANOVA multiple post hoc comparisons (Bonferroni method) for scores of bgPC 1 and 2 according to extant primates genera. (PDF) [file pone.0091944.s001.pdf]

Table S1. Significance for the ANOVA and MANOVA multiple post hoc comparisons (Bonferroni method) for scores of bgPC 1 and 2 according to extant primates genera.

	Cebus	*Ateles*	*Colobus*	*Cercopithecus*	*Papio*	*Hylobates*	*Symphalangus*	*Pongo*	*Pan*	*Gorilla*
**bgPC1**										
Ateles	*	-								
Colobus	*	***	-							
Cercopithecus	**	***	NS	-						
Papio	NS	***	NS	NS	-					
Hylobates	***	*	***	***	***	-				
Symphalangus	***	***	***	***	***	NS	-			
Pongo	***	**	***	***	***	NS	NS	-		
Pan	***	*	***	***	***	NS	NS	NS	-	
Gorilla	***	NS	***	***	***	NS	*	NS	NS	-
Homo	NS	NS	***	***	***	***	***	***	***	*
**bgPC2**										
Ateles	NS	-								
Colobus	NS	NS	-							
Cercopithecus	NS	NS	NS	-						
Papio	NS	NS	NS	NS	-					
Hylobates	NS	NS	NS	NS	NS	-				
Symphalangus	**	NS	NS	*	***	NS	-			
Pongo	*	***	***	**	NS	***	***	-		
Pan	NS	*	*	NS	NS	**	***	NS	-	
Gorilla	*	***	***	***	*	***	***	NS	NS	-
Homo	***	***	***	***	***	***	***	NS	NS	NS
**MANOVA (bgPC1 and bgPC2)**										
Ateles	*	-								
Colobus	*	***	-							
Cercopithecus	**	***	NS	-						
Papio	NS	***	NS	NS	-					
Hylobates	***	*	***	***	***	-				

Symphalangus	***	***	***	***	***	NS	-			
Pongo	***	**	***	***	***	NS	NS	-		
Pan	***	*	***	***	***	NS	NS	NS	-	
Gorilla	***	NS	***	***	***	NS	*	NS	NS	-
Homo	NS	NS	***	***	***	***	***	***	***	*

Abbreviations: bgPC, between-group principal component; NS: not significant; *, $p < 0.05$; **, $p < 0.005$; ***, $p < 0.001$.
